# Supplementary material for: Selection on modifiers of genetic architecture under migration load
Source: PLoS Genet. 2022 Sep 7;18(9):e1010350. doi: 10.1371/journal.pgen.1010350 (PMC9484686; doi:10.1371/journal.pgen.1010350)
Supplement: S1 Appendix — Appendix A contains an alternative formulation for the models that uses matrix and tensor notation to represent the population genetic recursion equations. Appendix B relates the current model to models of the spread of inversions under migration selection balance. (PDF) [file pgen.1010350.s001.pdf]

Supplemental Information 1 Appendix, from Selection on modifiers of genetic architecture under migration load

Stephen R. Proulx[1*], Henrique Teotónio[2],

1 Department of Ecology, Evolution, and Marine Biology, UC Santa Barbara, Santa Barbara, California, United States of America.
2 Institut de Biologie, École Normale Supérieure, Paris, France.

* stephen.proulx@gmail.com

Appendix A: Alternative life-cycle algebra

The life-cycle of the ancestral resident population can be described using tensor notation. This framework is useful as it can be modified to include complexities, such as non-Mendelian inheritance, transgenerational carryover environmental effects, etc., which the recursion equations presented in the main text cannot.

Given that there are k bi-allelic loci and random mating, the 2^k possible haplotypes are followed. The allele values are defined as 0/1 and the haplotypes are indexed by their binary numerical value offset by one, such that $0, 0, 0$ maps to 1, and 0,0,1 maps to 2, etc. Haploid population frequency in deme j is presented as:

$$\vec{h^j} = \begin{bmatrix} p_1^j \\ p_2^j \\ \vdots \\ p_{2^k}^j \end{bmatrix}$$

where p_i^j represents the frequency of the ith haplotype in the jth deme.

The diploid population frequency matrix is then formed by taking the outer product of $\vec{h}$ with itself, i.e. multiplying the frequency of all haplotype pairs in all possible orders:

$$\mathbf{d^j} = \vec{h^j} \otimes \vec{h^j},$$

with migration then mixing the densities between demes:

$$\mathbf{dm^j} = \mathbf{d^j}(1-m) + \mathbf{d^{\sim j}}(m),$$

where $\sim j$ inverts the binary value of j, and therefore represents the index for the other deme.

Diploid individual fitness matrix is defined as $W^j_{(h_1,h_2)}$ is the fitness of an (h_1, h_2) individual in deme j and has dimension $[2^k, 2^k]$:

$$\mathbf{W^j} = \begin{bmatrix} w^j(1,1) & w^j(1,2) & \cdots & w^j(1,2^k) \\ w^j(2,1) & w^j(2,2) & \cdots & w^j(2,2^k) \\ \vdots & \vdots & \ddots & \vdots \\ w^j(2^k,1) & w^j(2^k,2) & \cdots & w^j(m,n) \end{bmatrix}$$

where $w^j(x, y)$ is the fitness function in deme j for an individual carrying haplotypes x and y.

After selection, we re-normalize the vector of genotype frequencies by dividing by population mean fitness which ensures that the genotype frequencies sum to 1.

The diploid frequency after migration and selection is then:

$$\mathbf{dms^j} = \frac{\mathbf{dm^j} \circ \mathbf{W^j}}{\bar{w^j}},$$

where we use $\circ$ to represent element-by-element matrix multiplication (i.e. the Hadamard product); the mean fitness in deme j being $\bar{w^j}$.

The next stage of the life-cycle is gamete production where meiotic segregation and recombination of parental haplotypes takes place. Gamete production is accomplished by Hadamard tensor multiplication, which means that each diploid genotype frequency is multiplied by the vector of recombination probabilities of producing each gamete haplotype. For example, in a one locus scenario the gamete production vector is $v = [1/2, 1/2]$, so if the frequency of a heterozygote is x then the product is $x * v = [x/2, x/2]$. The gamete production tensor is a rank 3 tensor of length 2^k in each dimension, giving

$$(d \to h)^j = \mathbf{G} \circ \mathbf{dms^j},$$

where now $(d \to h)^j \in \Re^{2^k x 2^k x 2^k}$ where $(d \to h)^j_{(i1,i2,i3)}$ represents the frequency of type $i3$ haplotypes produced by parents with diploid genotype $(i1, i2)$. While $(i1, i2)$ and $(i2, i1)$ parents are identical in all ways, they appear as separate entries for computational convenience. And the total frequency of haplotypes is found by summing up the terms for a specific haplotype, that is, $\sum_{i3}(d \to h)^j_{(i1,i2,i3)}$. This can be represented in tensor operations by first unfolding, or flattening, $(d \to h)^j$ by a single level and then by taking the inner product with a one's vector.

Having described the life-cycle of the resident population we compare the notation presented in the main text with the tensor notation above. The ancestral population will attain a population genetic equilibrium between migration, selection, mating, segregation, recombination and syngamy, after iterating:

$$x_i^{d\prime} = \sum_j \sum_k w^d_{j,k} \left(x^d_j x^d_k (1 - m_d) + \sum_{\hat{d} \neq d} x^{\hat{d}}_j x^{\hat{d}}_k m_{\hat{d}} \right) G_{i,j,k} \quad (1)$$

$$\bar{w}^d = \sum_i x_i^{d\prime} \quad (2)$$

$$x_i^{d\prime\prime} = \frac{x_i^{d\prime}}{\bar{w}^d}, \quad (3)$$

where x^d_i is the frequency of haplotype i in deme d, $w^d_{j,k}$ is the fitness of genotype (j, k) in deme d, $G_{i,j,k}$ represents the probability that an adult with haplotypes j and k will produce a gamete of haplotype i, and $\bar{w}^d$ is the average fitness within deme d. Haplotype frequency in the next generation is given by $x_i^{d\prime\prime}$.

With tensor notation these recursion equations can be represented as:

$$d^{j\prime} = (\vec{h^j} \otimes \vec{h^j})(1 - m_j) + \sum_{i \neq j} (\vec{h^i} \otimes \vec{h^i})(m_i) \quad (4)$$

$$d^{j\prime\prime} = \frac{d^{j\prime} \circ \mathbf{W^j}}{\bar{w^j}} \quad (5)$$

$$h^{j\prime} = \sum_{i1} \sum_{i2} G \circ d^{j\prime\prime} \quad (6)$$

where equations 1, 2, 3, are analogous to equations 4, 5, 6 respectively.

Appendix B: Inversions and epistasis modifiers

We wish to understand the spread of inversions, eliminating recombination between local adaptation loci, as a function of selection on modifiers of epistasis. For this, we first write several definitions to simplify notation.

The frequency of haplotypes carrying i locally maladapted alleles is in deme d defined as $x_{(d)(i)}$. Define the average fitness among haplotypes that do not disperse (residents) from deme d as $\overline{W}_{HR(d)} = \sum_i x_{(d)(i)}(1-S)^{i/(2k)}$, and the average fitness among haplotypes that do disperse (migrants) as $\overline{W}_{HM(d)} = \sum_i x_{(d)(i)}(1-S)^{(k-i)/(2k)}$. The diploid average fitness is in deme d is $\overline{W_{D(d)}} = \overline{W_{HR(d)}}^2(1-m_d) + \overline{W_{HM(\sim d)}}^2 m_{\sim d}$, where $\sim d$ represents "not deme d", i.e. the other deme. We then define the fitness load among non-dispersing haplotypes as $L_{R(d)} = 1 - \overline{W_{HR(d)}}$, and the fitness load among dispersing haplotypes as $L_{M(d)} = 1 - \overline{W_{HM(d)}}$. The total haploid load is defined as $L_d = L_{R(d)}(1-m_d) + L_{M(\sim d)} m_{\sim d}$.

The invasion matrix for the inversion is given by

$$A_{\text{inv}} = \begin{vmatrix} \frac{\overline{W_{HR(1)}}(1-m_1)}{\overline{W_{D(1)}}} & \frac{\overline{W_{HM(1)}}m_1(1-S)^{1/2}}{\overline{W_{D(2)}}} \\ \frac{\overline{W_{HM(2)}}m_2}{\overline{W_{D(1)}}} & \frac{\overline{W_{HR(2)}}(1-m_2)(1-S)^{1/2}}{\overline{W_{D(2)}}} \end{vmatrix} \tag{7}$$

Define λ_{inv} as the dominant eigenvalue of A_{inv} which is the discrete time growth factor, i.e. R_0, for the inversion.

With unidirectional migration from deme 2 to 1, the matrix reduces to a lower diagonal matrix with an eigenvalue of $\lambda = \frac{\overline{W_{D(1)}}}{\overline{W_{D(1)}}^2 + m_2}$. We define the selection coefficient for the inversion as $s_{\text{inv}} = \lambda_{\text{inv}} - 1$, which is approximately $s_{\text{inv}} = L - m$, which is equation 4 from [20]. Note that this equation is quite general, it does not depend on any assumptions about the number of loci. It stems from the simple fact that the inversion haplotype has constant marginal fitness set to 1 and that the frequency of the inversion haplotype is reduced by m each generations (see also [31] for a general derivation of this result).

Under bi-directional migration with symmetry between the demes, we can find the eigenvalues and then approximate around weak selection. Under symmetry, we suppress the deme indices. We first Taylor expand around weak load, and then Taylor expand around small S, including second order terms (supplementary Mathematica file). We found that our approximation was both qualitatively and quantitatively accurate when we include the second order terms in S, but fails qualitatively when we only include the first order terms. Defining $s_{\text{inv}} = \lambda - 1$, we have

$$s_{\text{inv}} = L - \frac{S}{4}(L+1) - \frac{S^2}{8}(L + 1 - \frac{1}{4m}(L_M + 1)) \tag{8}$$

It is difficult to gain much intuition directly from this expression.

We develop a similar approximation for a complete modifier of epistasis, using the simplifying assumption that the modifier is always in a haplotype that contains at least one allele that is locally adapted in deme 1. This gives the matrix:

$$A_{\text{epi}} = \frac{1}{\overline{W_D}} \begin{vmatrix} (1-m) & m(1-S) \\ m & (1-m)(1-S) \end{vmatrix} \tag{9}$$

Approximating around weak load and weak S, and dropping terms of order $S^2 * L$ we come to:

$$s_{\text{epi}} = 2L - \frac{S}{2}(2L+1) - \frac{S^2}{4}(1 - \frac{1}{2m}) \tag{10}$$
